# Prevalence and associated factors of anemia among pregnant women of Mekelle town: a cross sectional study

**DOI:** 10.1186/1756-0500-7-888

**Published:** 2014-12-09

**Authors:** Abrehet Abriha, Melkie Edris Yesuf, Molla Mesele Wassie

**Affiliations:** Department of Human Nutrition, College of Medicine and Health sciences, University of Gondar, Gondar, Ethiopia

**Keywords:** Anemia, Prevalence, Associated factors, Pregnant, Women

## Abstract

**Background:**

Nutritional anemia is the most common type of anemia worldwide and mainly includes iron, folic acid, vitamin B_12_ and vitamin C deficiencies. Anemia is a global public health problem affecting people in all age groups but the burden of the problem is higher in pregnant women. The study aimed to assess prevalence of anemia and associated factors among pregnant women attending antenatal care in governmental health institutions in mekele town.

**Methods:**

Institution based cross-sectional study was employed. Systematic random sampling procedure was employed to select 619 study subjects. Pretested questionnaire were used to collect the data. The predictive value of the variable to Anemia was identified by bivariate and multiple logistic regression analysis.

**Result:**

The overall prevalence of anemia among pregnant women was 19.7%. Meal frequency less than two per day [AOR 3.93 95% CI (2.0,7.9)], Low Dietary Diversity score [AOR 12.8 95% CI (6.4,25.6)], Medium Dietary Diversity score [AOR 2.4 95% CI (1.2,4.8)], Parity [AOR 2.3 95% CI (1.4,3.8)] and Meat consumption less than once per week [AOR 2.2 95% CI (1.0,4.9)] were found to be factors affecting Anemia in pregnant women.

**Conclusion:**

Anemia among pregnant women is found to be mild public health problem in the study area. Parity, meal frequency, dietary diversity and meat consumption were significantly and independently affect anemia of pregnant women. Using family planning methods and improved meat consumption contributes for decreasing prevalence of anemia. Moreover, Diversifying food intake and increasing meal frequency of pregnant women is highly recommended.

## Background

Anemia is affecting 1.62 billion people globally [1]. The prevalence of anemia in developing countries is estimated to be 43% and that of developed countries is 9%. Anemia is estimated to contribute to more than 115 000 maternal deaths and 591 000 prenatal deaths globally per year [2]. Anemia occurs at all stages of the life cycle but its risk is higher in state of pregnancy due to an increased iron requirement, physiological demand, loss of blood and due to infections [1, 3].

Nutritional anemia is the most common type of anemia worldwide and mainly includes iron, folic acid, vitamin B_12_ and vitamin C deficiencies [1, 3–5]. Iron deficiency contributes for half of the burden of anemia globally [6]. Iron deficiency affects 1.3 to 2.2 billion persons out of those 50% are women of reproductive age [7]. In Ethiopia nearly 17% of women with age 15–49 are anemic of these 22% are pregnant women [8].

The contextual factors contributing for anemia among pregnant women are different. Interaction of multiple factors like women’s’ socio-demographic, socio- economic, nutritional and health related factors cause anemia in pregnant women. There is no adequate information on factors leading to anemia in pregnant women in Ethiopia and Mekele town in particular. Hence this study aims to provide evidence-based estimates of the magnitude and associated factors of anemia among pregnant women attending ANC in Mekelle governmental health institutions in the town.

## Methods

Facility based quantitative cross-sectional study was employed from February to April, 2014 at Mekelle town which is located at a distance of 783 km from Addis Abeba (capital of Ethiopia). The total number of women with in reproductive age was 68,093. Among these 11,011 were pregnant. There were five Governmental Hospitals, nine health centers and 4 private hospitals in mekele town and surrounding community which were giving ANC service in the study period [9].

### Study population

All pregnant women attending ANC in governmental health institutions of Mekelle town were target for the study. All pregnant women who attend ANC for the first time in the selected governmental health institutions during the data collection period were included in the study. Pregnant women who were seriously ill during the data collection period and pregnant women with repeated visits were excluded from the current study.

### Sample size and sampling

Sample size was determined by taking prevalence of anemia in a study done on Shalla woreda which was 12% [10], with 5% marginal error, design effect of 2, 95% CI and a non response rate of 10%. Based on this assumption, the final sample size was 632. Multistage sampling was employed to select pregnant women. Two Governmental hospitals and five health centers were selected randomly by using lottery method. The average number of pregnant women who visit ANC in health institutions in one month time was obtained by referring the client registration books to calculate the sampling interval which was 3. The calculated sample size were used to recruit study subjects from health Institutions proportional to their size. Finally study subjects were selected by using systematic random sampling technique.

The questionnaire, which was administered in the local language included questions that assessed socio-economic and demographic factors, pregnancy related characteristics, dietary diversity and meal frequency.

Blood Hemoglobin level was used to assess anemia status of pregnant women and women with <11 g/dl of blood hemoglobin level were considered as anemic. Meal frequencies for selected food items were asked for their habit one week prior to data collection. Dietary diversity score (DDS) was calculated by gathering information on dietary intake using single 24 hour dietary recall method. The score was categorized as Low (DDS ≤ 3), medium (DDS = 4 or 5) and high (DDS ≥ 6). Pregnant women taking at least one additional meal per day in addition to regular meal were considered as having good meal frequency. Value 0 was given for those having anemia and 1 for with no anemia in the analysis.

### Data collection tools, procedures and data quality management

Interviewer administered questionnaire was used to collect information about pregnant women. Data on hemoglobin level were collected by reviewing charts of pregnant women.

Seven nurse data collectors and two supervisors were trained for one day before the actual data collection. The structured questionnaire were translated in to Tigrigna (local language) and retranslated back to English to ensure accuracy of translation in to Tigrigna language. Questionnaire were pre-tested in Quiha health center which includes 32 pregnant women. After the pretest questions, ambiguous words or anything wrong was corrected before the final questionnaire is printed and distributed.

### Data Processing and Analysis

Data were entered in to Epi Info version 3.5.3 and analyzed using SPSS version 20 statistical software. Proportion and summary statistics was done to describe the study participants in relation to relevant variables. Both Bivarate and multivarate analysis were carried out. Variables with p value less than 0.2 in Bivariate analysis were entered in to multivariate logistic regression model. Variables P value less than 0.05 were taken as statistically significant and adjusted odds ratio with 95% CI was considered to see association.

### Ethical Consideration

The proposal was reviewed and approved by the Institutional Review Board (IRB) of University of Gondar. Health institutions were communicated and permission was obtained to proceed on the study. After the purpose and objective of the study have been informed, verbal informed consent were obtained from each pregnant women included in the study. Participants were informed as participation is on voluntary basis. In order to keep confidentiality of any information the data collection procedure was anonymous.

## Result

### Socio-demographic characteristics

A total of 632 respondents were included in the study with 97.9% response rate. The mean age of the respondents was 27.4 ± 5.5 years. More than two third of the respondents were married and 484 (78%) were orthodox Christian followers. Majority (85.1%) of the respondents were Tigre in ethnicity followed by Amhara 41 (6.6%). Majority of respondents were urban dwellers (88.2%) and 262 (42.3%) were housewife. Majority of the respondents were unable to read and write 223 (36%), 224 (36.2) were in the age group 26–30 and 215 (34.7%) of respondents earned (Table 1).

**Table 1:** **Socio-demographic and economic characters of pregnant women attending Antenatal care in Mekelle town, Northern Ethiopia, 2014**

Variables	Frequency (N = 619)	Percentage
**Age category**		
16-20	67	10.8
21-25	188	30.4
26-30	224	36.2
31-35	64	10.3
36-40	76	12.3
**Religion**		
Orthodox	484	78.2
Catholic	40	6.5
Muslim	76	12.3
Protestant	19	3.1
**Marital status**		
Married/live together	489	79.0
Divorced/separated/widowed	130	21.0
**Ethnicity**		
Tigray	527	85.1
Amhara	41	6.6
Afar	34	5.5
Oromo	17	2.7
**Residence**		
Urban	546	88.2
Rural	73	11.8
**Educational status**		
Unable to read and write	223	36.0
Read and write	187	30.2
Primary education	112	18.1
Secondary and above education	97	15.7
**Occupational status**		
Housewife	262	42.3
Private employ	113	18.3
Government employee	172	27.8
Farmer	72	11.6
**Family Monthly Income**		
Low level	145	23.4
Low Meddle	215	34.7
High Meddle	125	20.2
High level	134	21.6

### Pregnancy related Characteristics

**Figure 1:**
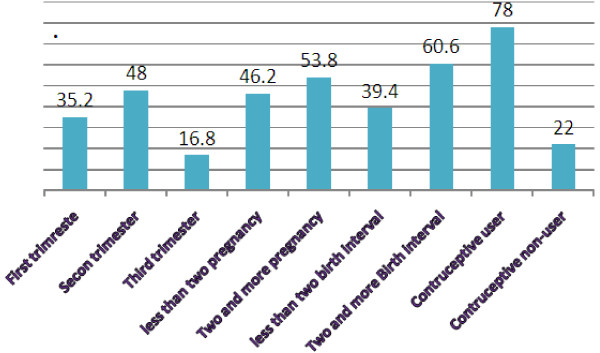
**Pregnancy related characteristics of pregnant women attending ANC in Mekelle town, Northern Ethiopia, 2014.**

Two hundred ninety-seven of the respondents were in the second trimester pregnancy. More than half (53.8) of the respondents were with parity of two and above, and 78% pregnant women were using contraceptive prior to current pregnancy (Figure 1).

### Nutritional Characteristics

Injera and wet was the staple diet for 418 pregnant women. Around half of the pregnant women ate three times per day. More than half of pregnant women (57.8%) took meat once per week, about 210 (33.4%) of women took milk twice per week. Two hundred eighty three (45.7%) of the respondents reported that they took egg twice per week. similarly around half of pregnant women ate fruits once per week. Majority of pregnant women took vegetables twice per week 452 (73%), two hundred seventy one (43.8%) of pregnant women were with medium dietary diversity score. The mean dietary diversity score of the respondents were 4.9 (Table 2).

**Table 2:** **Dietary Characteristics of pregnant women attending Antenatal care in Mekele town health institutions, Northern Ethiopia, 2014**

Variables	Frequency	Percentage
**Staple foods**		
Injera and wet	418	67.5
Maize and Sorghum	155	25.0
Spagati and Rice	46	7.4
	619	100%
**Meal Frequency**		
More than three times per day	126	20.4
Three times per day	318	51.4
Less than two times per week	175	28.3
**Frequency of taking Milk**		
More than twice per day	140	22.6
Once per day	127	20.5
Once per week	108	17.4
Twice per week	210	33.9
More than twice per week	34	5.5
**Frequency of taking Egg**		
Once per week	173	27.9
Twice per week	283	45.7
More than twice per week	163	26.3
**Frequency of taking Fruit**		
Once per week	311	50.2
Twice per week	197	31.8
More than twice per week	111	17.9
**Frequency of taking vegetable**		
Once per week	87	14.1
Twice per week	452	73
More than twice per week	80	12.9
**Dietary Diversity Score**		
Low	142	22.9
Medium	271	43.8
High	206	33.3
**Nutritional status**		
<21 cm	49	7.9
21 cm-23 cm	230	37.1
>23 cm	340	54.9

### Prevalence of Anemia

The mean ± SD hemoglobin concentration was 11.7 g/dl ± 2.32 and an overall prevalence rate of anemia with hemoglobin level < 11 g/dl was 19.3% (CI:19.1, 19.5). In terms of severity, mild anemia was 13.7%, moderate anemia was 4.4% and severe anemia was 1.6%.

## Discussion

Anemia is found to be a mild public health problem in the study groups. This finding is consistent with study conducted in Gondar town and Nine regional states of Ethiopia with the prevalence of 21.6% [11] and 18% [12] respectively. The result of this study was lower than the previous studies done on pregnant women at ANC clinic in Shalla Worda, in Urban Pakistan, in rural Uganda, in rural Vietnam and Ghana [3, 13–16] but higher than a study done in Iran and Awassa where the prevalence was found to be 13.1% and 15% respectively [17, 18].

Socioeconomic and geographical variations may be the reasons for different prevalence's of anemia in pregnant women across countries. Using different cutoff points for anemia may also resulted varied prevalence of anemia.

Multiple logistic regression analysis revealed that number of pregnancy, Meal frequency, Dietary diversity and frequent consumption of meat were significantly associated with anemia at p-value ≤ 0.05. Age category, Family monthly income, Marital status and occupational status of pregnant women showed significant association by bivariate analysis but not on the multivariate analysis (Table 3).

**Table 3:** **Factors affecting anemia among pregnant women in Mekelle town, Northern Ethiopia, 2014**

Variable	Anemia		Crude OR (95% CI)	AOR (95% CI)
	Yes	No		
**Number of pregnancy**				
< 2	38 (12.3%)	248 (87.70%)	1	1
≥ 2	84 (25.2% 0)	249 (74.8%)	2.20 (1.44,3.35)	2.38 (1.44, 3.94)*
**Trimester**				
First Trimester	30 (13.8%)	188 (86.2%)	1	
Second Trimester	65 (22%)	232 (78%)	1.75 (1.09,2.81)	
Third Trimester	27 (26%)	77 (74%)	2.19 (1.22,3.93)	
**Meal Frequency**				
>3 times per day	15 (12%)	111 (88%)	1	1
3 times per day	31 (9.8%)	287 (90,2%)	0.79 (0.41,1.53)	0.56 (0.27,1.17)
≤2 times per day	76 (43.4%)	99 (56.6%)	5.68 (3.06,10.52)	3.88 (1.93,7.79)
**Frequency of Taking meat per week**				
Once per week	93 (26%)	265 (74%)	2.42 (1.27, 4.64)*	2.23 (1.01,4.94)
Twice per week	17 (10.2%)	149 (89.8%)	0.78 (.36, 1.73)	0.53 (0.20, 1.38)
More than twice per week	12 (12.8%)	83 (87.2%)	1	1
**Dietary Diversity Score**				
Low	69 (48.6%)	73 (51. 4%)	12.03 (6.47,22.37)	12.82 (6.42, 25.62)*
Medium	38 (14%)	233 (86%)	2.07 (1.10, 3.89)	2.42 (1.22, 4.79)*
High	15 (7.2%)	191 (92.8)	1	1

*Those variables showing significant association in the multivariate analysis.

Note: Backward stepwise LR method was used to select factors. The model adequately fits the data at p-value = 0.198 (Hosmer and Lemeshow goodness of fit test).

Pregnant women with lower level of Dietary diversity score were around 13 times more likely to develop anemia than those with higher dietary diversity score. This finding is consistent with a study done in nine regional states of Ethiopia [12]. Studies conducted in Pakistan and Turkey also suggested consumption of fruit two or more times per week is associated with a decreased risk of anemia [14, 19]. Poor dietary diversity leads to deficiency of minerals and vitamins which may increase bio-availability of iron then affects Iron status [20]. Pregnancy is the most nutritionally demanding period in a woman’s life. Consequently, pregnant women are advised to eat more diversified diet than usual [12] *.*

Consumption of meat were also another factor which showed significant association with Anemia in pregnant women. Pregnant women with habit of eating meat once per week were 2.2 times at higher risk of developing anemia than pregnant mothers who ate meat more than twice per week. This finding is consistent with other studies in which pregnant women who ate red meat two or more times a week had higher mean hemoglobin concentrations [6, 12, 14, 15, 19]. The increased concentration of hemoglobin is with the fact that red meat is an important source of heme iron [10, 21].

The present study also identified that, the odds of repeated pregnancies more than two or more were 2.3 times greater among pregnant mothers as compared to those who have less than two number of pregnancies. This result is consistent with the study done in Pakistan [14].This is due to the fact that Short intervals between births may not provide women with enough time to replenish lost nutrient stores before another reproductive cycle begins [22]. The risk is considerably exacerbated in those conditions where balanced diets is not available [5].

Pregnant women who had meal frequency less than two times per day were 3.9 times at higher risk of developing anemia than those whose meal frequency was more than three times per day. This might be due to the fact that pregnancy is a special period with increased energy and nutrient requirement which can be fulfilled with increased meal frequency.

### Limitation of the study

There will be a recall and/or social desirability bias while subjects were requested to give dietary information and monthly income. Exclusion of patients with severe anemia may lower the prevalence in the study groups. Moreover, cross sectional nature of the study limits measuring the cause and effect relationship.

## Conclusion

Anemia is found to be a mild public health problem in the study area. Number of pregnancy, Meal frequency, Food diversity and frequent consumption of meat were variables affecting anemia in pregnant women. Awareness creation on contraceptive use, nutritional counseling on consumption of iron-rich foods and Iron/foliate supplementation are recommended to prevent anemia in pregnant women.

## Abbreviations

ANC: Antenatal care
AOR: Adjusted odds ratio
CI: Confidence interval
COD: Crude odds ratio
DDS: Dietary diversity score
EDHS: Ethiopia demographic health survey
Hgb: Hemoglobin
IDA: Iron deficiency anemia
MUAC: Middle upper arm circumference
OR: Odds ratio
SPSS: Statistical package for social science
WHO: World Health Organization.

## References

[1] McLean E, Cogswell M, Egli I, Wojdyla D, de Benoist B (2009). Worldwide prevalence of anaemia, WHO vitamin and mineral nutrition information system, 1993–2005. Public Health Nutr.

[2] Balarajan Y, Ramakrishnan U, A–zaltin E, Shankar AH, Subramanian SV (2013). Anaemia in low-income and middle-income countries. Lancet.

[3] Mbule MA, Byaruhanga YB, Kabahenda M, Lubowa A, Mbule M (2013). Determinants of anaemia among pregnant women in rural Uganda. Rural Remote Health.

[4] Edward B (2010). Regular vitamin C supplementation during pregnancy reduces hospitalization: outcomes of a Ugandan rural cohort study. Pan Afr Med J.

[5] Abdelrahman EG, Gasim GI, Musa IR, Elbashir LM, Adam I (2012). Red blood cell distribution width and iron deficiency anemia among pregnant Sudanese women. Diagn Pathol.

[6] Haidar JA, Pobocik RS (2009). Iron deficiency anemia is not a rare problem among women of reproductive ages in Ethiopia: a community based cross sectional study. BMC Blood Disord.

[7] Saeed AAA, Asif A, Zulfiqar A, Muhammad R, Tariq I (2013). Iron status of the Pakistani population-current issues and strategies. Asia Pac J Clin Nutr.

[8] CSA [Ethiopia] and ORC Macro (2011). Ethiopian Demographic and Health Survey.

[9] Office Mekelle Zonal Health (2012). Health Management Information System Anuual Report.

[10] Obse N, Mossie A, Fau-Gobena T, Gobena T: **Magnitude of anemia and associated risk factors among pregnant women attending antenatal care in Shalla Woreda, West Arsi Zone, Oromia Region, Ethiopia.** 1029–1857 (Print) PMC3742894 23950633

[11] Meseret Alem BE, Aschalew G, Tigist K, Mohammed S, Olkeba Y (2013). Prevalence of anemia and associated risk factors among pregnant women attending antenatal care in Azezo Health Center Gondar town, Northwest Ethiopia. J Interdiscip Histopathol.

[12] Gebremedhin S, Enquselassie F (2005). Correlates of anemia among women of reproductive age in Ethiopia: evidence from Ethiopian DHS. Ethiopian J Health Dev.

[13] Obse N, Mossie A, Gobena T (2013). Magnitude of anemia and associated risk factors among pregnant women attending antenatal care in Shalla Woreda, West Arsi Zone, Oromia Region, Ethiopia. Ethiopian J Health Sci.

[14] Baig-Ansari N, Badruddin SH, Karmaliani R, Harris H, Jehan I, Pasha O, Moss N, McClure EM, Goldenberg RL (2008). Anemia prevalence and risk factors in pregnant women in an urban area of Pakistan. Food Nutr Bull.

[15] Ritsuko Aikawa NCK, Satoshi S, Binns CW (2006). Risk factors for iron-deficiency anaemia among pregnant women living in rural Vietnam. Public Health Nutr.

[16] Amengor MG OW, Akanmori BD (2005). Determinants of anemia in pregnancy in Sekyere West district Ghana. Ghana Med J.

[17] Barooti E, Rezazadehkermani M, Sadeghirad B, Motaghipisheh S, Tayeri S, Arabi M, Salahi S, Haghdoost A (2010). Prevalence of iron deficiency anemia among Iranian pregnant women; a systematic review and meta-analysis. J Reprod Infertil.

[18] Gies SBB, Yassin MA, Cuevas LE (2003). Comparison of screening methods for anemia in pregnant women in Awassa Ethiopia. Trop Med Int Health.

[19] Karaoglu L, Pehlivan E, Egri M, Deprem C, Gunes G, Genc MF, Temel I (2010). The prevalence of nutritional anemia in pregnancy in an east Anatolian Province, Turkey. Health.

[20] Jemal HNH, Urga K (1999). Iron deficiency anemia in pregnant and lactating mothers in rural Ethiopia. East Afr Med J.

[21] Antelman G, Msamanga GI, Spiegelman D, Urassa EJN, Narh R, Hunter DJ, Fawzi WW (2000). Nutritional factors and infectious disease contribute to anemia among pregnant women with human immunodeficiency virus in Tanzania. J Nutr.

[22] Government of the Federal Democratic Republic of Ethiopia (2013). National Nutrition Programme.

